# Keep an Eye on Next Generation Sequencing (NGS) Technology: Secondary Findings and Differential Diagnosis in Inherited Retinal Dystrophies (IRDs)

**DOI:** 10.3390/biomedicines13051117

**Published:** 2025-05-05

**Authors:** Fabiana D’Esposito, Matteo Capobianco, Caterina Gagliano, Alessandro Avitabile, Giuseppe Gagliano, Gabriella Esposito, Edoardo Dammino, Antonio Carotenuto, Marco Zeppieri

**Affiliations:** 1 Imperial College Ophthalmic Research Group (ICORG) Unit, Imperial College, London NW15QH, UK; f.desposito@imperial.ac.uk; 2 Department of Neurosciences, Reproductive Sciences and Dentistry, University of Naples Federico II, 80131 Napoli, Italy; 3 Department of Ophthalmology, Catania University San Marco Hospital, 95121 Catania, Italy; 4 Department of Medicine and Surgery, University of Enna “Kore”, Piazza dell’Università, 94100 Enna, Italy; 5 Mediterranean Foundation “G.B. Morgagni”, 95100 Catania, Italy; 6 Department of Molecular Medicine and Medical Biotechnologies, University of Naples Federico II, 80100 Naples, Italy; 7 CEINGE-Advanced Biotechnologies Franco Salvatore, 80100 Naples, Italy; 8 Department of Ophthalmology, University Hospital of Udine, 33100 Udine, Italy; 9 Department of Medicine, Surgery and Health Sciences, University of Trieste, 34127 Trieste, Italy

**Keywords:** next generation sequencing NGS, NGS incidental findings, NGS secondary findings, inherited retinal dystrophies, genotype–phenotype correlation, molecular diagnosis

## Abstract

**Background**: Next Generation Sequencing (NGS) Technology has represented a revolution in the molecular characterization of Inherited Retinal Dystrophies (IRDs), which are among the most genetically and phenotypically heterogeneous conditions. NGS has allowed the characterization of a consistent number of patients affected by IRDs, but at the same time, unexpected results can pose diagnostic dilemmas. **Aim**: The purpose of this review is to describe possible scenarios as a reference for ophthalmologists and geneticists who are involved in this particularly complex field. **Methods**: A review of the existing literature has been performed. In addition, examples have been brought, from a series of patients that have been analyzed at the University of Naples “Federico II”-CEINGE Biotecnologie Avanzate “Franco Salvatore”. **Results**: Unexpected results in the genetic characterization of IRDs are not uncommon. The main findings are additional variants that potentially modify phenotypes, deletions masked by apparent homozygosity, and pathogenic variants leading to phenotypes revisitation. **Conclusions**: The high genetic and phenotypic heterogeneity characterizing IRDs have been greatly advantaged by the advent of NGS Technology. At the same time, the not uncommon finding of unexpected data poses diagnostic criticisms that need to be addressed. In this review, we describe possible scenarios, and we go through some more complex genotype–phenotype correlations.

## 1. Introduction

Inherited Retinal Dystrophies (IRDs) encompass a large and heterogeneous group of pathologies characterized by a progressive degeneration of the retinal photoreceptors (cones and/or rods) and the retinal pigment epithelium, resulting in progressive deterioration of the visual function, with variable degrees of progression and severity [1,2]. IRDs display a considerable phenotypic heterogeneity that, coupled with an extremely high genetic heterogeneity, make the molecular diagnosis crucial for the correct definition of the pathological entity in each patient [3]. At present, through Next Generation Sequencing (NGS) techniques, large amounts of nucleic acid fragments can be analyzed in parallel and simultaneously, with excellent speed, yield, and precision, through different programs of specific panels of genes, coding regions (Whole Exome Sequencing or WES), or Whole Genome Sequencing (WGS) testing. Through NGS, not only the identification of variants in known genes has been made fast and efficient, but new disease-related genes and mechanisms have been identified [4,5].

The identification of an increasing number of IRD-related genes has been made possible by the wide application of NGS technology for the genetic characterization of patients worldwide, leading to increasingly accurate and rapid diagnoses, and the ambitious possibility to register patients into databases, aiming at potential access to innovative and increasingly personalized gene therapies [1,6,7,8]. Thanks to the wide application of WES and WGS, the genetic landscape of IRDs has been more comprehensively defined, and syndromic forms, previously underdiagnosed or unacknowledged, have also been characterized [8,9,10,11]. The increasing availability of more efficient genetic tests through NGS has in fact made it possible to achieve molecular diagnostic rates close to 60–70% in some reports [1,10,12]; however, a significant proportion of patients still remain without a definitive molecular diagnosis. This occurs in most cases in relation to the presence of deep intronic variants, structural defects, such as copy number variants (CNVs) and/or rearrangements and oligogenic forms of inheritance [2,6,11].

On the basis of these considerations, improving diagnostic capacity can also lead to advances towards feasibility of therapeutic options: in this respect, gene therapies are emerging as a possible strategy, such as the administration of subretinal recombinant viral vectors (voretigene neparvovec) for the treatment of patients affected by IRDs related to biallelic *RPE65* pathogenic variants [13,14,15]. Moreover, other strands of research are being investigated, either based on genetic supplementation with other than *RPE65* genes constructs with viral vectors, or genome editing platforms such as CRISPR/Cas9, with the aim of ‘correcting’ the defective gene [2,13,16]. Other innovative approaches include the use of stem cells and optogenetic approaches in order to rescue functional transmission capacity in the degenerated retinal structures [17,18,19]. However, these innovative approaches are not yet optimized and need further studies and investigations to improve the safety profile, reduce production costs, and evaluate possible long-term complications [18,20].

Although NGS techniques are considered fast and efficient in the perspective of patients genotyping, the interpretation of the results can be difficult and non-univocal in some cases. As techniques and technologies improve, the researcher is exposed to a greater amount of data emerging from the tests, posing the challenge of a correct interpretation of potential multiple genetic defects [21,22]. The final interpretation is often challenging, in relation to the presence of variants of uncertain significance (VUS) and incidental ‘secondary findings’ [1,8,12]. The correct interpretation of such findings cannot be separated from the multidisciplinary framing of the patient with the collaboration of geneticists, ophthalmologists, bioinformatics and bioethics experts, in order to correctly integrate the laboratory data with the clinical and instrumental aspects [6,11,23].

Despite the fact that considerable progress has been made, there are still many challenges to be faced: high costs limit access to these technologies for a large number of patients, with significant differences among countries [24,25], the difficulty in interpreting results, especially in the presence of VUS variants and incidental additional findings [26], the translational gaps between research and clinical-diagnostic-therapeutic implementation [2,12], and the management of genetic information with the ethical implications this information may have for patients and their families [27,28]. The future challenges will focus on the optimization of the previously mentioned approaches, the improvement of diagnostic methodologies (e.g., long-read sequencing or optical genome mapping techniques), and a better understanding of the genetic data at our disposal, VUS and incidental secondary findings [29,30].

In this narrative review we describe a selection of possible scenarios emerging from NGS genetic testing in genotype–phenotype correlations, particularly focusing on situations where the molecular diagnosis has addressed different strategies in the management of patients and in genetic counseling. Three illustrative clinical cases are also presented, describing how the diagnostic pathway and the interpretation of data actually takes place.

## 2. Materials and Methods

The review was conducted by performing a literature search on the main databases (PubMed, Medline, the Cochrane Library and ClinicalTrials.gov) using keywords such as ‘Inherited Retinal Diseases’, ‘Retinitis Pigmentosa’, ‘Stargardt Disease’, ‘Usher Syndrome’, ‘Next Generation Sequencing’, etc. Only publications in English were considered. The various selected articles were analyzed, and the extrapolated data integrated into the drafting of this review. In particular, studies describing the use of WES, WGS, and gene panels were considered, accompanied by details on the bioinformatic analysis pipeline. The classification of genetic variants was preferably based on the criteria of the American College of Medical Genetics (ACMG), classifying variants into pathogenic, probably pathogenic, of uncertain significance (VUS), probably benign, or benign [31]. As far as the ethics committee was concerned, this being a narrative review, no approval was required, but consent for the publication of results for scientific purposes was given by those patients and/or their guardians who underwent genetic testing. In this study, we included systemic sclerosis patients with varying durations of disease, resulting in a heterogeneous sample ranging from relatively recent diagnoses (two years after diagnosis) to patients in the chronic phase of the disease (more than 20 years). We then proceeded to analyze ophthalmological data and disease characteristics, ultimately correlating these parameters. This type of study, despite ocular vascular involvement not being one of the most frequent ocular complications in patients with systemic sclerosis, has proven useful for obtaining information on the prevalence of a condition or a specific characteristic within our population at a precise moment.

In this narrative review, we present three illustrative clinical examples retrospectively selected from patients who have previously received genetic counseling and diagnostic testing at our institution. These instances did not need fresh patient recruitment or prospective data collection, and all participants or their legal guardians have granted written informed consent for the scientific utilization of their anonymized data, in compliance with institutional ethical standards.

## 3. Results

With a view to an increasingly integrated and multidisciplinary approach, the literature review and case reports confirm the value of a diagnosis based on the results emerging from NGS techniques and the importance of proper management, integration, and interpretation of the available data. Next-generation sequencing is widely used to uncover the genetic causes of Inherited Retinal Dystrophies [32]. NGS can simultaneously screen the entire set of known IRD-related genes (gene panels), it can perform a systematic analysis of the coding regions of all genes (Whole Exome Sequencing), or it can sequence the entire content of a patient’s genome (Whole Genome Sequencing). However, despite continuous development in NGS technology, pathogenic variants are detected in approximately 60% of IRD cases [1]. This can be partially explained by the fact that most evaluations of IRD-related genetic variants are focused on small insertions and deletions or single-base substitutions (SNVs), while the molecular background of Inherited Retinal Dystrophies is far more complex and heterogenous, including deep-intronic sequence changes, mutations in regulatory regions, chromosomal rearrangements with loss or gain of genes/exons, and copy number variations (CNVs) [33,34].

NGS technology is an indispensable tool to clarify unresolved cases of IRDs; however, it may also unveil secondary or unexpected findings which call in to question initial diagnosis or lead to phenotypes revisitation.

### 3.1. Variants Potentially Modifying Phenotypes

**Figure 1:**
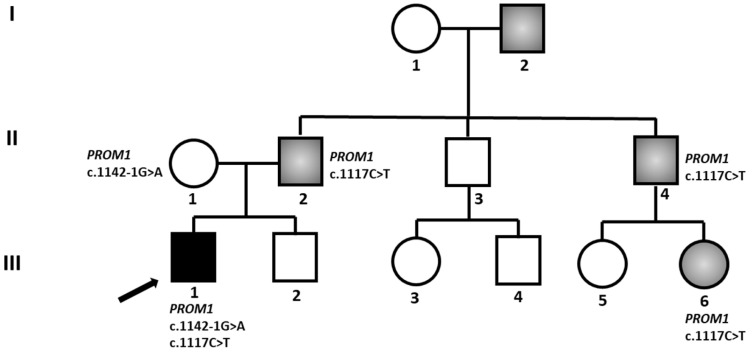
Patient 1 Pedigree. Squared boxes indicate males, circles indicate females, closed symbols represent affected, and open symbols represent unaffected persons. Roman numbers define the generation and arab numbers the individuals. Different scales of gray/black indicate different severity, with the gray shade corresponding to a milder phenotype. The arrow indicates the proband. The identified variants are indicated next to tested individuals.

We present the case of a patient (Patient 1), providing an example of how a second allele can modulate the phenotypic expression of an IRD. The proband was a male aged 22, manifesting visual impairment since pre-scholar age. At observation, his visual acuity (VA) was hand motion (HM) in both eyes (OU). In his clinical history, he reported nyctalopia in the first phases, alongside with photophobia and progressive visual decay. No other significant systemic features were present. In his family history, he reported his father, paternal uncle, grandfather, and cousin (Figure 1) being diagnosed with Stargardt macular dystrophy, but with no genetic testing being carried out. The patient’s parents were studied, and the father, aged 51, displayed macular dystrophy, far less severe than his son, with visual acuity decay beginning in his third decade, and at our observation being 6/9 in OU. The proband’s mother did not show any sign of retinal dystrophy. The other family members were not seen by us but reported to have a similar phenotype to the father. No consanguinity was present in the different generations of the pedigree, and the family did not originate from a genetic isolate.

Beside the clinical aspect, the dominant pattern of transmission was not consistent with the *ABCA4* suspected gene [35] and the genetic investigations confirmed the exclusion. With the NGS/WES technique, two class 5 pathogenic variants in the *PROM1* gene were identified, namely c.1117C>T and c.1142–1G>A. *PROM1* encodes the protein promin-1, implicated in the morphological genesis of photoreceptor disks: pathogenic variants in promin-1 are, therefore, correlated with macular and cone-rod dystrophies, displaying both dominant and recessive modes of inheritance [36]. Segregation study of the two variants in the proband’s parents confirmed compound heterozygosity, with variant c.1117C>T being dominantly inherited through the family generations, and variant c.1142–1G>A being inherited from the mother, who was unaffected, confirming the recessive behavior of this variant.

In these patients, the clinical and genetic characterization of the proband’s parents was of fundamental importance as it not only allowed us to define the genetic causes of his pathology but also confirmed the hypothesis that additional variants in a gene in a compound heterozygous state can modulate the severity of the phenotype, explaining the different clinical severity in our proband and his affected family members [36]. Furthermore, this example highlights the importance of carrying out extensive sequencing strategies and techniques, not focusing on a single ‘candidate’ gene, as variants with a modifying effect can further, and randomly, be found in genes less commonly associated with IRD.

### 3.2. Deletions Masked by Apparent Homozygosity

In recessive diseases, subjects presenting the same variant in homozygosis could be classified as ‘homozygous’ in a basic genetic analysis and that single variant could be considered responsible for the pathologic phenotype. However, sometimes, a structural variant in the opposite allele can occur, giving the effect of an ‘apparent homozygosity’ in the sequence pattern. Taking as an example an individual that has inherited a pathogenic variant on one allele and, on the other, an exonic or multiexonic deletion, if the bioinformatics equipment has not been adequately set to detect CNVs, it could classify the affected region as identical to the one with the allelic mutation (since the other corresponding allele is physically missing). A paradoxical result will, therefore, be obtained in which the subject will appear to possess the same allelic variant in homozygosis when, in reality, the genetic defect is a compound heterozygosis with a point variant and an extended deletion on the other allele [37,38]. This is not rare in IRDs, where the high number of recessive genes increases the possibility of encountering such situation [36,39,40]. Proper interviewing about possible consanguinity and familial segregation analysis of identified variants, alongside with CNV detection strategies, is mandatory when a homozygous pathogenic variant is reported. Awareness about this possibility is crucial, not only for a correct molecular diagnosis, but also for genetic counseling-related issues.

### 3.3. Pathogenic Variants Leading to Phenotypes Revisitation

A further element to evaluate is the case in which the identification of disease-related variants lead to a re-evaluation of the underlying condition, unveiling a syndromic form. For example, at our center, we evaluated a little girl aged 2.5 years (Patient 2), manifesting alternating exotropia with a dominant right eye (OD) and an albinoid fundus in absence of extraocular signs of albinisms. Both parents and her two brothers displayed a normal fundus and visual function. Genetic testing resulted in the presence of two variants in compound heterozygosis in the *HPS5* gene: c.100C>A (p.Arg34Ser), predicted as being a VUS, and c.219G>A (p.Arg73=), predicted as likely pathogenic. The *HPS5* gene has been associated with Hermansky–Pudlak syndrome (HPS), characterized by oculocutaneous albinisms (with variable manifestations), bleeding diathesis, and, in some individuals, pulmonary fibrosis, granulomatous colitis, and/or immunodeficiency. Cutaneous hypopigmentation can be so irrelevant that some individuals (like our patient) can be mistakenly diagnosed with ocular albinism [41]. Following the results of genetic testing, an accurate anamnesis has been recorded, and the parents have reported a tendency of bleeding in the child, which had not been investigated before. The little patient underwent all the necessary evaluations to investigate the syndromic features of her condition, and beside the hematological defect, did not display any other sign of HPS.

### 3.4. Phenotypic Consequences of High Levels of Consanguinity and Unexpected Preclinical Diagnosis

**Figure 2:**
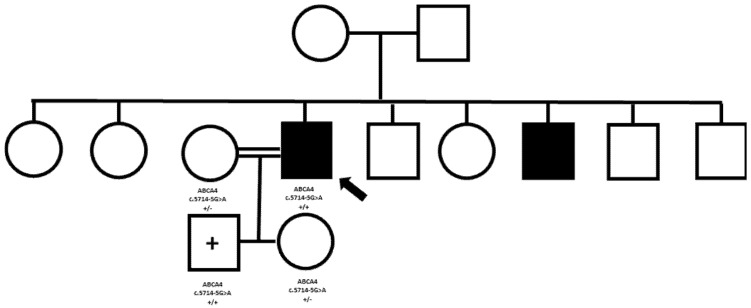
Patient 3 Pedigree. Squared boxes indicate males, circles indicate females, closed symbols represent affected, and open symbols represent unaffected persons. A ‘+’ sign indicates a genetically affected but clinically still unaffected patient. The arrow indicates the proband. The identified variants are indicated next to tested individuals. The “−“ minus sign indicates a wild-type allele.

We describe the case of a male patient aged 43 (patient 3), with a clinical diagnosis of Stargardt macular dystrophy, supported by electrodiagnostic testing and multimodal imaging [42]. The patient originated from a small, isolated village in Albania, and reported affected brothers and unaffected parents (Figure 2). WES genetic testing revealed a homozygous pathogenic variant in the *ABCA4* gene (c.5714-5G>A). Real homozygosity was likely to be real, in consideration of the origin from a genetic isolate, but the proband’s parents were reported as non-consanguineous. Therefore, we decided to proceed to familial segregation analysis, to confirm the NGS result. Unfortunately, being our proband based in Italy, none of his family members was available to travel from Albania. We, therefore, decided to test his two children, a boy aged 8 and a girl aged 6, in order to confirm the presence of the variant in a heterozygous state. The unexpected result was that the son, at present unaffected, was homozygous for the pathogenic variant. At this point, a focused interview with the patient revealed a known level of consanguinity between the patient and his wife, who then proved to be heterozygous for the variant. This case highlights the importance of careful interviewing when defining a pedigree, in search of possible high levels of consanguinity, that can potentially give the impression of dominant transmission.

These case-based insights have highlighted the necessity for a more comprehensive contextual understanding, which we examine further through a cohesive analysis of existing literature.

## 4. Discussion

The findings detailed in the Results section, through specific clinical examples, have established a practical foundation for contemplating bigger concepts articulated in the literature. This presentation incorporates literature findings to contextualize the clinical complexity in our patients and to emphasize new diagnostic problems and genotype–phenotype interpretations.

Complex gene interactions may determine complex phenotypic traits, such as the ‘triallelic inheritance model’ described for Bardet–Biedl Syndrome [43,44]. Bardet–Biedl syndrome (BBS) is an autosomal recessive disease characterized by progressive retinal dystrophy, post-natal obesity, intellectual disability, hypogonadism, and post-axial polydactyly [45,46,47]. BBS exhibits genetic and phenotypical heterogeneity. Secondary clinical findings such as diabetes insipidus, auditory and speech impairment, asthma, and congenital heart disease may also be present with great variability, even in the same family [46]. To date, 25 BBS genes have been discovered [48,49]. Variants in TMEM67/MKS3 can modify BBS phenotypes in patients carrying pathogenic variants in other BBS genes [50]. Some BBS families were identified as carrying three variants in genes at two different BBS loci, segregating with variable expression of the phenotype [44]. The modifier effect of a third variant in a different genes is not surprising in BBS ciliopathy, as several BBS proteins may interact in a protein complex, in the same or complementary pathways [51]. cone and cone-rod dystrophies (CORDs) are one of the most common and heterogeneous Inherited Retinal Dystrophies [52].

To date, around 51 genes involved in phototransduction and photoreceptor morphogenesis have been linked to CORDs [48,53]. Most of their causative variants are inherited as an autosomal recessive trait, while variants in PRPH2, CRX, GUCY2D, GUCA1A, and PROM1 are usually associated with dominant forms, but with selected variants acting recessively. Pathogenic variants in RPGR, CACNA1F, OPN1LW, and OPN1MW genes account for X-linked forms of CORDs [48,53]. However, many other CORD-associated genes are still undetected. Moreover, precise genotype–phenotype correlations in CORDs are difficult to determine, because most genes are associated with uncertain and heterogenous phenotypes [54].

The presence of modifier genes and multigenic inheritance patterns can hinder definite molecular diagnoses and genotype–phenotype correlations [54]. Heterogeneity may be explained by different factors. Firstly, each gene may present various disease-related variants, leading to the same phenotype. Secondly, the different mutated genes can induce the same pathological phenotype. Thirdly, different mutations in the same gene can lead to different clinical scenarios, even in members of the same family [55].

Donato et al., in 2022, described a Sicilian family with four affected individuals presenting with clinical features consistent with cone-rod dystrophy, such as night blindness, dyschromatopsia, and progressive loss of visual field and best corrected visual acuity [56]. A supplementary diagnostic test showed undetectable ERG and prominent atrophic macular lesion in OCT [56]. A dominant inheritance pattern with a possible incomplete penetrance in the last generation was identified in the family. The causative variant proven to be related to the CORD onset was c.2513G>C in the GUCY2D gene.

The c.3044-7G>T variant in GUCY2D, commonly detected in affected patients, could probably exert a modifier role. However, the highly heterogenous phenotypes of affected family members could not be explained just by the presence of these two variants. Therefore, the authors focused on other genes that could act as modifiers, affecting the phenotypic and molecular expression of previously cited genes. Thus, nine variants in six genes (PAX2, RXRG, CACNG8, CCDC175, PDE4DIP, and LTF) were identified as potential modifiers. The only variant with a different distribution between affected and healthy subjects was CACNG8 c.6819A>T. Therefore, authors stated that the causative gene was GUCY2D, which encodes a retina-specific guanylate cyclase, which is highly expressed in the outer segment membranes of cones and rods [57].

The combination of c.2513G>C and c.3044-7G>T variants contributed to the main phenotype of family members. However, such cone compromised activity, caused by GUCY2D defects, might be differentially worsened by altered retrograde signaling from the inner retinal cells due to variants carried by modifier genes. The c.6819A>T variant in the CACNG8 gene proved to be the main modifier. Pathogenic variants in CACNG8, which encodes the γ-8 member of transmembrane AMPA receptor-associated regulatory proteins (TARPs), impairs interaction of photoreceptors with dopaminergic amacrine cells and retinal ganglion cells, reducing both retrograde and anterograde signal transmissions [56].

Next Generation Sequencing techniques can sometimes solve diagnostic dilemmas or call into questions previous diagnosis. In 2024, Alzahem et al. described an interesting clinical case of a 38-year-old female who had been previously diagnosed with Usher syndrome based on finding variants in three genes (MYO7A, USH2A, and PCDH15). She presented bilateral subnormal vision, acquired hearing loss, asymmetric retinopathy, and right macular pseudocoloboma. She also presented atrophy of the calf spaces and myopathic facies. Her clinical case was re-evaluated and Whole Exome Sequencing resulted in the detection of a variant in the PRPS1 gene (NM_002764.4:c.287G>A; p.Arg96Gln). The final diagnosis of PRPS1-associated retinopathy and Charcot–Marie–Tooth disease type 5 was made [58].

In Usher syndrome (USH) and other ciliopathies, accessory variants in other genes can modify phenotype expression due to protein interactions. USH is a group of autosomal recessively inherited dystrophies characterized by bilateral sensorineural hearing loss and retinal degeneration, with additional vestibular impairment in type I [59]. Schneider et al., in 2009, identified an additional causative gene for USH: the PDZD7 gene, which encodes a protein interacting with proteins encoded by USH1C and DFNB31, and it contributes to phenotypical heterogeneity of USH [60].

Inconsistency between supposed inheritance patterns and the genetic test results is common when testing IRDs with NGS [61,62]. For example, Zenteno et al. described a family with a sibship of six females and six males, in which four males were affected by Usher syndrome, suggesting a possible X linked inheritance (never previously described in USH). However, NGS revealed a homozygous pathogenic variant in USH2A, leading to a redefinition of the inheritance pattern as autosomal recessive [63]. In another family with a suspect case of sporadic cone-rod dystrophy, NGS identified a heterozygous dominant frameshift variant in the GUCA1A gene, leading to the identification of lack of penetrance in the transmitting father [63].

Extensive molecular analysis, including NGS, is fundamental in challenging families with complex pedigrees. Chen et al. demonstrated coexistence of different inheritance modes and mutations affecting causative genes in patients affected by Retinitis Pigmentosa (RP), belonging to two families with high levels of consanguinity [61]. The first family included two siblings who were born from inbred parents. However, no pathogenic variant were found being shared by both patients. Further analysis found that the female patient had a recurrent homozygous C8ORF37 p.W185*, while the male patient carried hemizygous OFD1 p.T120A [61]. The second family included two patients born to consanguineous parents who carried homozygous p.R419W variant in the TULP1 gene, while other four patients presented a recurrent heterozygous RP1 p.L762Yfs*17n with an autosomal dominant inheritance pattern [61]. Jones et al. described three emblematic families with complex pedigrees and demonstrated that caution should be taken when attributing an inheritance pattern or a single gene dis-ease-causing variant to an entire family [62].

For example, they described a family with four affected individuals and one presumed non-penetrant member. Genetic testing identified a dominant pathogenic variant in RP1 (p.Arg677Ter) that was carried by two of the four affected individuals but absent in the proband and the presumed non-penetrant individual. Further targeted NGS in the fourth affected individual demonstrated compound heterozygous pathogenic variants in USH2A (p.Cys419Phe, p.Glu767Serfs*21). In another family with 3 affected individuals and 1 presumed non-penetrant member, NGS identified three retinal dystrophy genes (PRPF8, PRPH2 and USH2A) with dis-ease-causing variants in varying combinations among the affected individuals. Genetic testing of family 3, composed of 7 affected individuals, identified a pathogenic variant in PRPH2 (p.Pro216Leu) consistent with disease in six of the seven affected individuals. Additional retinal NGS testing determined that the proband also carried a multiple exon deletion in the CRX gene likely accounting for her cone-rod phenotype; her son only carried the CRX variant, but not the familial variant in PRPH2 [62].

Identification of a disease-causing variant with a clear inheritance pattern in a proband may not be sufficient for targeted, known variant analysis in other family members. Families with phenotypic variation or apparent non-penetrant individuals may offer a clue to suspect complex in-heritance cases and to re-evaluate initial diagnosis [62].

Di Iorio et al. demonstrated that pseudodominant pattern of inheritance can hide an autosomal recessive Retinitis Pigmentosa due to copy number variations (CNVs). They described an Italian RP family characterized by EYS gene-related pseudodominant inheritance. The female proband, her brother, and both her sons showed typical signs of RP. To investigate this apparently autosomal dominant pedigree, NGS and bioinformatic detection CNVs were performed. Unexpectedly, all patients carried a compound heterozygosity involving two known pathogenic EYS variants, the exon 33 frameshift mutation c.6714delT, and the exon 29 deletion c.(5927þ1_5928-1)_(6078þ1_6079-1)del, with the exception of the youngest son who was homozygous for the above-detailed frameshift mutation. The proband’s husband was a heterozygous healthy carrier of the same c.6714delT variant in exon 33 of the EYS gene. Therefore, a CNVs study is necessary in those pedigrees that remain genetically unsolved after the NGS or WES [64].

For a correct diagnosis it is, therefore, important that the investigation protocol includes CNVs, whether they are duplications, deletions, or complex rearrangements. It is also important to carefully review the data obtained from the NGS technique. In many laboratories, specific pipelines for the investigation of CNVs and/or software that analyze the reading depth of the exons (coverage) are used. This allows us to highlight any discrepancies that could be related to deletions and/or duplications and subsequently verify such discrepancy with complementary techniques (qPCR, MLPA, or targeted array-CGH analysis). Furthermore, a fundamental and essential role is played by genetic counseling, which, beyond the various techniques, always plays a pivotal role. In conclusion, possible masked deletions in regions of apparent homozygosity can explain some particular forms of IRD classified as atypical, with unusual gene transmission patterns.

Information about the presence of a pathogenic genetic variant in a genetic isolate is particularly important, in order to provide the community with the necessary tools for ad hoc genetic counselling. Another very delicate issue arising from this case is related to preclinical genetic diagnosis. While knowing the presence of a variant that will cause a pathological phenotype in a healthy child might be a difficult information to manage for the child’s parents, it could be beneficial in some respects. First, early intervention can be undertaken, especially for the very few forms where potential therapies are becoming a reality. In addition, the information about the presence of a disease-causing genetic defect may contribute to awareness in lifestyle and professional choices [65].

## 5. Conclusions

Genetic testing in IRDs is becoming an increasingly important tool for the molecular characterization of affected patients and their family members. The well-established NGS technology provides the great advantages of being fast, efficient, and providing wide information about underlying genetic defects. Nevertheless, the resulting data often pose complex diagnostic challenges and unexpected genotype–phenotype correlations. We provide an overview of the current knowledge in the interpretation of the most frequently encountered possible scenario, as a tool for professionals involved in the molecular diagnosis of patients affected by IRDs.

## Data Availability

Data are contained within the article.
